# Der chirurgische Umgang mit peripheren Nerven nach Extremitätenverlust

**DOI:** 10.1007/s00132-020-04032-1

**Published:** 2020-11-24

**Authors:** Clemens Gstoettner, Gregor Laengle, Stefan Salminger, Christopher Festin, Hannes Platzgummer, Oskar C. Aszmann

**Affiliations:** 1 Klinisches Labor für Bionische Extremitätenrekonstruktion, Universitätsklinik für Chirurgie, Medizinische Universität Wien, Währinger Gürtel 18–20, 1090 Wien, Österreich; 2 Klinische Abteilung für Plastische und Rekonstruktive Chirurgie, Universitätsklinik für Chirurgie, Medizinische Universität Wien, Wien, Österreich; 3 Univ. Klinik für Radiologie und Nuklearmedizin, Medizinische Universität Wien, Wien, Österreich

**Keywords:** Amputation, Extremitätenprothesen, Bionik, Neurom, Phantomschmerz, Amputation, Artificial limbs, Bionics, Neuroma, Phantom limb pain

## Abstract

**Hintergrund:**

Nach Verlust einer Gliedmaße ist es die Aufgabe des Chirurgen, einen möglichst schmerzfreien und belastbaren Stumpf zu formen. Hierbei kommt insbesondere an der oberen Extremität ein funktioneller Aspekt hinzu, da zur Steuerung myoelektrischer Prothesen entsprechende Muskelsignale notwendig sind. Der Umgang mit peripheren Nerven im Stumpfbereich nimmt sowohl hinsichtlich der Schmerztherapie als auch der funktionellen Mensch-Maschinen-Anbindung eine zentrale Rolle ein.

**Ziel der Arbeit:**

Die Darstellung aktueller chirurgischer Verfahren zum Umgang mit peripheren Nerven nach Extremitätenamputation.

**Material und Methoden:**

Es erfolgt eine Literaturrecherche bzgl. chirurgischer Prophylaxe und Therapie von Neurom- und Phantomschmerzen, sowie zu Techniken zur Verbesserung der funktionellen Schnittstelle zwischen Stumpf und Prothese. Anhand relevanter Arbeiten sowie der Erfahrungen der Autoren werden entsprechende Empfehlungen formuliert.

**Ergebnisse und Diskussion:**

Es gibt eine große Anzahl an verschiedenen Operationstechniken, insbesondere im Umgang mit schmerzhaften Neuromen. Von den klassischen Verfahren findet besonders häufig die intramuskuläre Verlagerung der endständiger Nerven Anwendung. Neuere Techniken wie Targeted Muscle Reinnervation (TMR) und Regenerative Peripheral Nerve Interface (RPNI) zielen erstmals darauf ab, dem Nerven auch nach Amputation funktionelle Endorgane zu liefern. Neben der verbesserten Steuerung myoelektrischer Prothesen zeigen diese Verfahren auch exzellente Ergebnisse in Bezug auf Neurom- und Phantomschmerzen.

## Abstract (en)

**Background:**

After limb loss, it is the surgeon’s task to provide the patient with a pain-free and resilient residual limb. Particularly in the upper extremity, there is an additional functional aspect, as appropriate muscle signals are needed to control myoelectric prostheses. Surgical management of peripheral nerves within the residual limb plays a central role both in terms of pain treatment as well as functional human-machine interfacing.

**Objectives:**

The presentation of current surgical procedures for dealing with peripheral nerves after limb amputation.

**Material and methods:**

A literature search is carried out regarding the surgical prophylaxis and therapy of neuroma and phantom limb pain, as well as techniques to improve the functional interface between residual limb and prosthesis. Practical recommendations are formulated based on relevant literature, as well as the experiences of the authors.

**Results and conclusions:**

There is a large number of different surgical techniques, particularly for the management of painful neuromas. Of the conventional methods, intramuscular implantation of the terminal nerves is commonly used with good results. Newer techniques such as targeted muscle reinnervation (TMR) and the regenerative peripheral nerve interface (RPNI) aim for the first time to provide functional end organs to the nerve even after amputation. In addition to the improved control of myoelectric prostheses, these methods further show excellent results for treatment and prevention of neuroma and phantom limb pain.

Nach dem Verlust einer Extremität können periphere Nervenendigungen zu Missempfindungen und Schmerzen wie Phantom- oder Neuromschmerzen führen. Aktuelle Operationstechniken, wie die Targeted Muscle Reinnervation, können diesen peripheren Nervenendigungen reinnervierbare Ziele liefern und so Schmerzen verhindern. Diese Arbeit liefert einen Überblick über die grundlegenden Prinzipien im Management peripherer Nerven nach Amputation sowie eine detaillierte Beschreibung anerkannter Techniken.

## Einleitung

„The management of sectioned nerves remains a controversial aspect of amputation surgery. […] Numerous techniques have been devised to minimize neuroma formation, but none has proven uniformly successful.“

Douglas G. Smith, *Atlas of Amputations & Limb Deficiencies* (3rd Edition, 2004) [32]

Seit dieser Aussage hat die Amputationschirurgie verschiedene Entwicklungen durchlebt. Insbesondere mit der Targeted Muscle Reinnervation (TMR) wurde erstmals eine Operationstechnik präsentiert, welche peripheren Nerven nach Amputation ein reinnervierbares Ziel liefert. Diese und ähnliche Verfahren stehen heute den alten, konventionellen Methoden im Umgang mit peripheren Nerven gegenüber, allen voran der hohen Neurotomie und intramuskulären Verlagerung.

## Amputation – Funktionsverlust und Schmerz

Die traumatische oder elektive Amputation einer Gliedmaße bedeutet für die betroffene Person einen gravierenden Einschnitt in die Lebensqualität mit ausgedehnten psychosozialen Konsequenzen [3]. Während distale Amputationen etwa eines Fingergliedes oder einer Zehe für den Patienten im Alltag meist keine weitreichenden Folgen haben, steigt das Ausmaß der funktionellen Einschränkung sowie die Beeinträchtigung des Körperbildes mit der Höhe des Amputationslevels. Proximale Amputationen, wie etwa Exartikulation im Hüft- oder Schultergelenk, stellen eine besondere Herausforderung für den behandelnden Arzt und Orthopädietechniker dar. Der prothetische Extremitätenersatz gelingt in solchen Fällen selbst mit modernsten Mitteln meist leider nur unzureichend.

Während initial nach der Amputation, besonders bei ungeplantem, traumatischem Gliedmaßenverlust, die psychologische Betreuung an erster Stelle steht, wird der Patient im Verlauf einen Spezialisten aufsuchen, um eine bestmögliche Wiederherstellung des verlorenen Körperteils anzustreben. Hierbei kommt es zu enormen Unterschieden der Wünsche und Anforderungen verschiedener Patienten, welche nicht nur mit der Amputationslokalisation variieren, sondern auch stark von individuellen Faktoren wie Geschlecht, Alter, soziokulturellem Umfeld, Arbeit, Familienstand und nicht zuletzt Ausmaß und Art der amputationsbedingten Schmerzen beeinflusst werden. Letztere stellen nicht selten den primären Anlass zur ärztlichen Vorstellung dar. Bei vielen Patienten rückt daher der funktionelle Verlust aufgrund starker, andauernder Schmerzen in den Hintergrund.

Grundsätzlich wird bei amputationsbedingten Schmerzen zwischen Stumpf- und Phantomscherzen unterschieden. Zusätzlich können auch noch sekundäre Beschwerden im Bereich des unversehrten Bewegungsapparates auftreten, welche meist durch Fehlbelastung bedingt sind. Ein Beispiel hierfür sind etwa Rückenschmerzen nach Armverlust, bedingt durch Schiefhaltung der Wirbelsäule aufgrund der ungleichen Gewichtsverteilung [10]. Stumpfschmerzen entstehen häufig durch endständige Neurome der peripheren Nerven (siehe Abschnitt „Stumpfneurom“), welche einerseits durch Berührung und Bewegung gereizt werden, aber auch spontan Schmerzen verursachen können. Zur Behandlung von Neuromschmerzen wurde eine Vielzahl an chirurgischen Techniken vorgeschlagen und untersucht. Phantomschmerzen stellen hingegen ein schlecht verstandenes, komplexes Phänomen dar, dessen genaue Ursache nicht vollends geklärt ist. In der Literatur werden periphere wie auch zentralnervöse Faktoren postuliert. Besonders die funktionelle Reorganisation der kortikalen Areale, welche mit der amputierten Extremität in Verbindung stehen, scheint eine wichtige Rolle zu spielen [12]. Es gibt einige Behandlungsansätze, welche von konservativem Schmerzmanagement über verschiedene physiotherapeutische Maßnahmen bis hin zu modernen Methoden mithilfe von Virtual Reality reichen. Nicht zuletzt gewinnen aber auch chirurgische Interventionen an Stellenwert, bedingt durch positive Studienergebnisse der letzten Jahre [10, 20, 34].

Während Operationen an peripheren Nerven nach Amputation der unteren Extremität meist nur mit dem Ziel der Schmerzreduktion erfolgen, steht insbesondere bei Armverlust proximal des Ellenbogengelenkes auch der funktionelle Aspekt im Vordergrund. Dies ist dadurch bedingt, dass prothetischer Armersatz häufig mittels myoelektrischer Steuerung erfolgt, während Beinprothesen in der Regel ohne aktive Signalgebung durch den Benutzer auskommen. Da periphere Nerven im Stumpfbereich weiterhin kognitiv angesteuert werden können, ist es möglich durch selektive Transfers dieser Nerven auf die residuale Stumpfmuskulatur neue, intuitive Muskelsignale zur Prothesensteuerung zu schaffen (siehe Abschnitt „Targeted Muscle Reinnervation“). Der Verantwortung des behandelnden Chirurgen obliegt es somit, die spezifischen Anforderungen des jeweiligen Patienten zu erkennen und die bestmögliche Therapieoption entsprechend zu identifizieren.

## Das Stumpfneurom

**Figure 1:**
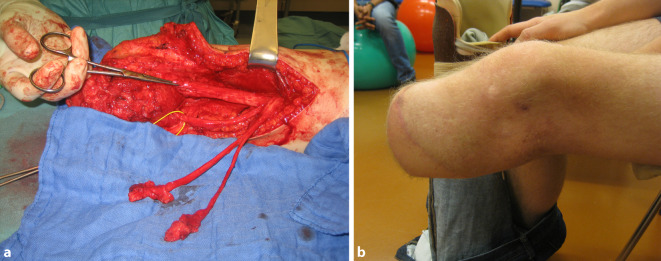

Bei Verlust einer Extremität verlieren periphere Nerven den Kontakt zu ihren Zielorganen. Nach traumatischer Durchtrennung eines Nervs wird proximal der Amputationsstelle ein komplexer Prozess eingeleitet, welcher das Regenerationspotenzial der Nervenzelle aktiviert [6]. Während das distale Axon durch Kontaktverlust zur Nervenzelle im Rahmen der Waller-Degeneration zersetzt und abgebaut wird, findet die Degeneration proximal meist nur sehr begrenzt statt, in der Regel bis zum nächsten Ranvier-Schnürring [24]. Im Verlauf beginnen die einzelnen Axone von proximal erneut auszuwachsen, auf der Suche nach einem entsprechenden Zielorgan. An der Spitze des regenerierenden Axons steht der sogenannte Wachstumskegel, ein spezialisiertes, bewegliches Gebilde, aus welchem mehrere fingerartige Fortsätze hervorgehen. Der Wachstumskegel ist verantwortlich für die Orientierung der Regeneration, was idealerweise in Richtung des distalen Nervenstumpfes erfolgen sollte. Das Wachstum orientiert sich an verschiedenen speziellen Stimuli, wie etwa neurotrophen Faktoren die in den distalen Schwann-Zellen produziert werden [24]. Des Weiteren bildet jedes einzelne Axon mehrere Kollateraläste aus, um die Chance auf erfolgreiche Regeneration zum ursprünglichen Zielorgan zu erhöhen. Da im Fall einer Amputation der distale Nervenstumpf jedoch verloren ist, finden die regenerierenden Axone in der Regel kein entsprechendes „Target“. Dadurch kommt es zu ungerichtetem Wachstum der Nervenfasern, was wiederum zur Ausbildung von Neuromen führt. Dies sind kolbenförmige Auftreibungen am Ende des Nervs, welche aus diffus angeordneten Axonbündeln sowie zu etwa 80 % aus narbigem Bindegewebe bestehen (Abb. 1; [35]) Es wurde nachgewiesen, dass die Nervenfasern innerhalb eines Neuroms sehr leicht erregbar sind und sogar spontan feuern können, was eine Erklärung für die resultierenden neuropathischen Schmerzen liefert [25, 37]. Grundsätzlich behält der Nerv jedoch sein regeneratives Potenzial, welches durch Schaffen einer frischen Läsion nach Resektion des Neuroms erneut aktiviert wird.

## Diagnostik

Die Diagnostik von Phantomschmerzen ist rein klinisch zu stellen. Patienten können diese in unterschiedlichen Schweregraden wahrnehmen, von einfachen und kurz anhaltenden Schmerzepisoden im fehlenden Körperteil bis hin zu konstanten und quälenden schmerzhaften Empfindungen [12]. Diese können z. B. als Stechen, Pochen, Brennen oder Krampfen beschrieben werden, mit Beginn direkt nach der Amputation oder auch erst nach mehreren Jahren.

**Figure 2:**
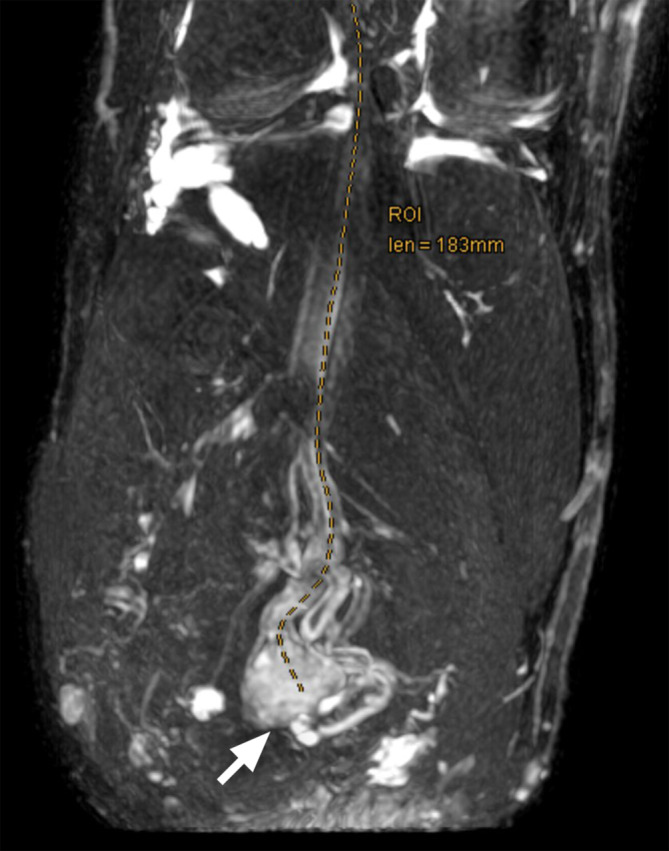

Der Verdacht eines schmerzhaften Stumpfneuroms ergibt sich initial auch klinisch, häufig aufgrund eines lokal auslösbaren Druckschmerzes am Stumpfende. Oft werden Patienten wegen dieser Problematik vorstellig, etwa nach Anpassung einer Prothese, welche durch Druck auf den Stumpf zu einer Exazerbation der Beschwerden führt. Der Amputierte kann die Lokalisation des schmerzauslösenden Punktes meist selbst gut identifizieren. Im Rahmen der Untersuchung wird durch Palpation bzw. Beklopfen des Stumpfes das Punctum maximum des Schmerzreizes identifiziert. Es ist dabei zu beachten, dass durchaus mehrere Neurome gleichzeitig Schmerzen bereiten können, bei transtibialen Amputationen etwa der N. peroneus communis und der N. tibialis. Im Sinne des Hoffmann-Tinel-Zeichens strahlt der Neuromschmerz bei Beklopfen in das distale sensible Versorgungsgebiet des Nervs aus, also beim N. peroneus communis in Richtung Fußrücken und beim N. tibialis in Richtung Fußsohle. Jedoch kann es Patienten auch schwerfallen, die genaue Lokalisation der Ausstrahlung zu benennen. In solchen Fällen wird auch nur von diffus schmerzhaftem Blitzen oder Kribbeln berichtet, welches auch ohne mechanischen Auslöser auftreten kann. Zur Feststellung der Lokalisation und des Ausmaßes von Stumpfneuromen ist bildgebende Diagnostik hilfreich. Grundsätzlich eignen sich sowohl Magnetresonanztomographie als auch hochauflösender Ultraschall zur Darstellung peripherer Nerven [18]. Bei der Magnetresonanzneurographie werden spezielle Sequenzen verwendet, wodurch sich Nervengewebe besonders gut visualisieren lässt (Abb. 2). Um ein Neurom endgültig als Schmerzauslöser zu identifizieren, kann eine Ultraschall-gezielte Blockade des betroffenen Nervs mittels Lokalanästhetikum sehr nützlich sein, was zu temporärer Schmerzfreiheit führen sollte.

## Nervenchirurgische Eingriffe am Stumpf

Es existiert kein allgemein akzeptiertes Standardvorgehen im Umgang mit peripheren Nerven im Rahmen von Amputationen bzw. dem Management schmerzhafter Neurome im Verlauf. Zwischen 150 und 200 verschiedene chirurgische Techniken zu Prävention und Therapie von Neuromen wurden in der Literatur beschrieben [35]. In den 1970er-Jahren wurde es noch empfohlen, Neurome als solche zu belassen und lediglich in narbenfreies und mechanisch unbelastetes Gewebe zu verlagern [8]. Es konnte jedoch tierexperimentell nachgewiesen werden, dass endständige Neurome sehr empfindlich gegenüber mechanischer Belastung sind und auch die Quelle spontaner, ektoper Signale sein können [25, 37]. Somit war es historisch eines der wichtigsten und bis heute geltenden Erkenntnisse, dass die Resektion eines schmerzhaften Neuroms notwendig für die chirurgische Therapie ist, wodurch eine frische Läsion weiter proximal geschaffen wird. Alle in Folge beschriebenen Techniken sind daher sowohl bei der primären Amputation, sowie auch sekundär bei bereits etablierten Stumpfneuromen anwendbar.

Die Resektion schmerzhafter Neurome ist der erste Schritt der chirurgischen Therapie

**Figure 3:**
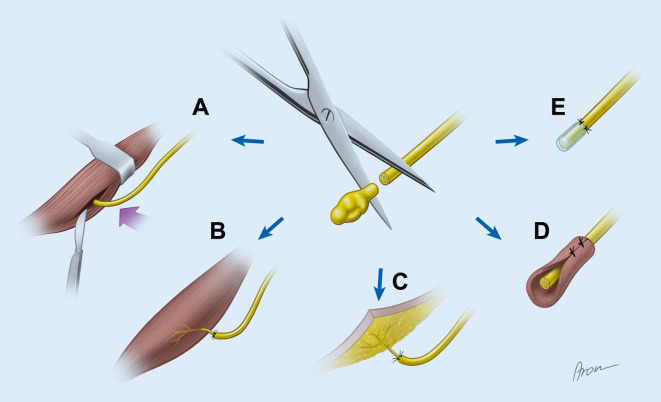

Häufig werden im Rahmen der primären Amputation Nerven unter Zug rückgekürzt, damit die Nervenenden proximal der terminalen Belastungszone des Stumpfes zu liegen kommen. Hierbei kommt es im Verlauf zur Bildung von Neuromen, welche dem Patienten Schmerzen verursachen können. Zur Behandlung von bereits bestehenden Neuromen ist eine alleinige Resektion nicht zu empfehlen, da eine Reoperation aufgrund wiederkehrender Beschwerden in bis zur Hälfte der Fälle notwendig sein kann [14]. Um die Neubildung von Neuromen zu verhindern, wurden verschiedene Techniken angewandt. Eine Rückkürzung der Faszikel und darauffolgende Ligatur der epineuralen Hülle konnte, verglichen mit alleiniger Neuromresektion, keine Verbesserung der Ergebnisse erzielen [17]. Bessere Erfahrungen wurden nach Verschluss der Nervenöffnung mittels einem epineuralen Patch berichtet, welcher zuvor vom resezierten, distalen Nerv entnommen wird [38]. Ein vom Prinzip ähnliches „capping“ des Nervs mittels Venen oder Silikonkappen (Abb. 3e) wurde vorgestellt, jedoch bisher ohne überzeugende Ergebnisse in größeren klinischen Studien [11]. Eine weitere, selten angewandte Technik ist die zentrozentrale Neurorrhaphie, bei der der Nerv longitudinal interfaszikulär getrennt wird und die entstehenden Nervenenden miteinander vernäht werden [2]. Außerdem wurde eine End-zu-Seit Nervennaht an gesunde Nerven vorgeschlagen, was im Tiermodell zu einer Prävention von Neuromen führte [1].

Die meiste klinische Erfahrung gibt es für die Verlagerung von Nervenenden in narbenfreies Gewebe. Hier wird insbesondere die intramuskuläre Verlagerung (siehe Abschnitt „Intramuskuläre Transposition“) angewandt, alternativ wurden auch Venen [26] oder Knochen [13] gewählt. Besonders die letztgenannte Technik findet in der Handchirurgie häufig Anwendung. Alle erwähnten Operationsmethoden haben gemeinsam, dass dem regenerierenden Nerven keine freien Endorgane zur Reinnervation geboten werden. Demgegenüber wurden rezent verschiedene, vielversprechende Techniken vorgestellt, welche auf Reinnervation von Muskel oder Haut im Stumpfbereich beruhen und somit auch von funktioneller Relevanz für die spätere Prothesenversorgung sind (siehe Abschnitte „Targeted Muscle Reinnervation“, und „Regenerative Peripheral Nerve Interface“).

### Intramuskuläre Transposition

Die vermutlich am Häufigsten angewandte Technik zur Therapie von Neuromen ist die Rückkürzung und Implantation von Nerven in einen naheliegenden Muskel, was auch als intramuskuläre Transposition bezeichnet wird (Abb. 3a). Im Jahr 1918 berichtete Ludwig Moszkowicz in Wien erstmals, das proximale Ende eines Nervs in einen innervierten Muskel implantiert zu haben, um eine wiederkehrende Neurombildung zu verhindern [8]. Dies war bei zwei Patienten erfolgreich. Dellon et al. haben 1984 in einer Arbeit zu diesem Thema experimentelle Ergebnisse an Pavianen präsentiert [9]. Während die Kontrollnerven nach Rückkürzung und Verbleib in der Subkutis ein Neurom mit Narbenbildung entwickelten, zeigte sich bei Nerven im Muskel keine klassische Neurombildung, bei Erhalt des parallelen Verlaufs der Nervenfasern und ohne wesentliche Narbenbildung. Klinisch wurde diese Technik für Neurome vieler verschiedener Nerven angewandt, mit weitgehend gutem Erfolg hinsichtlich Neuromschmerzen. Rungprai et al. konnten etwa zeigen, dass eine intramuskuläre Verlagerung zu einer signifikant höheren Schmerzreduktion als einfache Neurotomie bei Neuromen am Fuß führt [28]. In einem rezenten Review von Dellon und Aszmann wird von einer Erfolgsquote von zumindest 80 % für diese Technik ausgegangen [8]. Ein Versagen dieser Technik trete laut den Autoren dann auf, wenn ein Muskel mit großer biomechanischer Auslenkung für die Implantationsstelle ausgewählt wird, wenn ein kleiner Muskel zu nahe an der Haut für die Implantationsstelle verwendet wird oder wenn ein anderer, nicht erkannter Nerv ebenfalls Teil des Schmerzmechanismus ist.

### Targeted Muscle Reinnervation

Die Operationsmethode der Targeted Muscle Reinnervation wurde 2004 erstmals von Kuiken et al. an einem Patienten mit glenohumeraler Amputation vorgestellt [22]. Bei dieser Technik werden die großen Nerven des Armes (insbesondere N. medianus, N. ulnaris, N. radialis und N. musculocutaneus) selektiv auf die einzelnen motorischen Äste bzw. „motor entry points“ der residuellen Stumpfmuskulatur transferiert (Abb. 3b und 4). Somit kommt es zu einer Reinnervation dieser Muskeln durch Nerven, welche kognitiv mit den Funktionen des verlorenen Armes verknüpft sind. Der Patient kann nach erfolgreicher Reinnervation und Rehabilitation etwa bei Denken an Faustschluss selektiv den M. pectoralis minor kontrahieren. Dieses intuitive Muskelsignal kann dann zur Steuerung einer myoelektrischen Prothese verwendet werden. Bergmeister et al. konnten zeigen, dass sich bei TMR der Muskel an den Nerv anpasst, wodurch es zu einem Switch der Muskelfasertypen bzw. auch zu einer Hyper-Reinnervation kommen kann [4].

**Figure 4:**
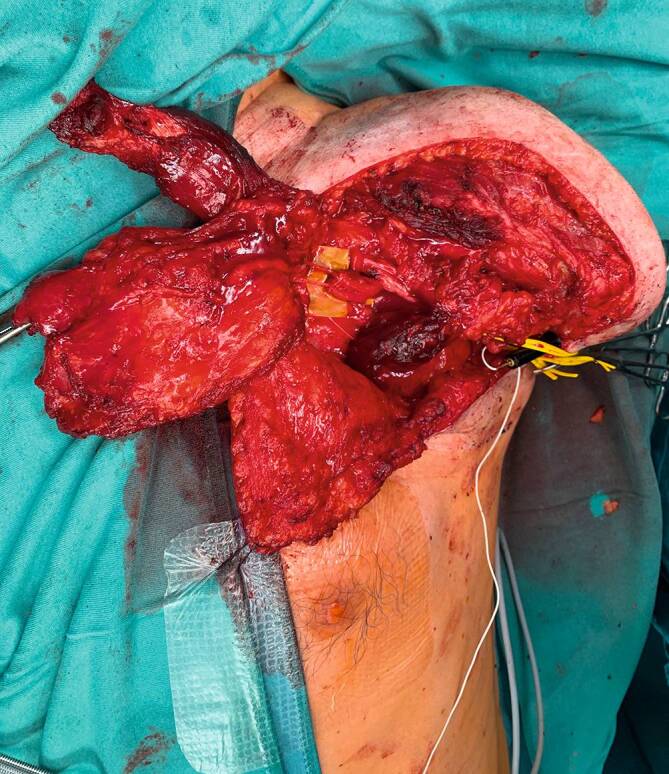

TMR führt zu einer Reduktion von Phantom- und Neuromschmerzen

Die detaillierte Operationstechnik wurde 2015 von Salminger et al. für glenohumerale sowie transhumerale Amputationshöhen beschrieben [29]. Rezent wurden funktionelle Ergebnisse nach 30 TMR-Operationen bei Patienten mit Amputation proximal des Ellenbogengelenkes präsentiert [30]. Bei weiter distalen Amputationen, etwa transradial, besteht kein Mismatch zwischen der Anzahl der zur Verfügung stehenden Signale und den notwendigen Freiheitsgraden der Prothesensteuerung, sodass TMR zu rein funktionellen Zwecken nicht regelmäßig angewandt wird. Da myoelektrische Prothesen an der unteren Extremität nur im Rahmen von Forschungsprojekten getestet werden, hat auch hier der funktionelle Aspekt der TMR keinen Stellenwert in der klinischen Praxis.

Auch wenn TMR ursprünglich zur verbesserten Prothesensteuerung bei hohen Amputationen der oberen Extremität konzipiert wurde, gibt es inzwischen eine Vielzahl an Publikationen zu den positiven Effekten dieser Technik auf Phantom- wie Neuromschmerzen nach Amputation. Von Dumanian et al. wurde 2018 erstmals eine prospektive, randomisiert-kontrollierte Studie zur Behandlung von Postamputationsschmerzen vorgestellt [10]. Bei 28 Patienten mit Majoramputationen und chronischen Schmerzen wurde entweder TMR oder Neuromexzision mit submuskulärer Verlagerung durchgeführt. Nach einem Jahr zeigte sich anhand der Numeric Rating Scale (NRS) eine signifikant höhere Reduktion der Phantomschmerzen in der TMR-Gruppe (−3,2 NRS) verglichen mit der Kontrollgruppe, wo sich kein eindeutiger Unterschied zur präoperativen Situation nachweisen ließ (+0,2 NRS). Ebenso erzielte TMR nach einem Jahr eine deutlichere Verbesserung der Stumpfschmerzen (−2,9 NRS vs. −0,9 NRS). Auch bei den finalen Nachuntersuchungen schnitt die TMR-Gruppe wesentlich besser ab. Der positive Effekt von TMR auf Postamputationsschmerzen wurde von den Autoren so begründet, dass der regenerierende Nerv neue Verbindungen mit den afferenten Rezeptoren in einem Muskel schließt, und somit die efferent-afferente Feedback-Schleife wieder vervollständigt wird. Dies ist auch mit dem Konzept der Deafferenzierung als Ursache für Phantomschmerzen vereinbar. Inzwischen wurde weiters eine Studie präsentiert, bei der TMR bereits während der initialen Amputation durchgeführt wurde. Auch hier zeigten sich signifikant bessere Ergebnisse bei Phantom- wie auch Neuromschmerzen im Vergleich zu einer Kontrollgruppe ohne spezifische Intervention [34]. Aufgrund dieser überzeugenden Ergebnisse wurde in den letzten Jahren die Indikationsstellung von TMR an verschiedenen Amputationshöhen der oberen wie unteren Extremität untersucht und die entsprechenden Operationstechniken detailliert beschrieben [5, 7, 21, 27].

Ein interessanter Nebeneffekt der TMR wurde schon von Kuiken et al. beobachtet, nämlich dass ein gemischt motorisch-sensibler Nerv nicht nur den Muskel reinnerviert, sondern nach Entfernung des subkutanen Fettgewebes auch die darüberliegenden Hautareale [22]. So wird nach Reinnervation durch den N. medianus auch die Haut über dem jeweiligen Muskel sensibel mit Medianusaxonen versorgt und vermittelt bei Berührung entsprechende Empfindungen der Hand. Derselbe Effekt wird auch mittels Targeted Sensory Reinnervation (TSR) verfolgt, mit dem Unterschied, dass der Nerventransfer hier nicht auf einen Muskelast, sondern direkt auf einen sensiblen Nerven vollzogen wird (Abb. 3c; [15]). Inzwischen wurde mittels funktioneller MRT gezeigt, dass bei Patienten nach reinnervierenden Eingriffen am Stumpf die Aktivität der primären motorischen und sensiblen Kortexareale denen von gesunden Patienten ähnelt, im Gegensatz zu Amputierten ohne solche Interventionen [31]. Dies deutet darauf hin, dass diese reafferenzierenden Eingriffe pathologische kortikale Anpassungen nach Amputation vorbeugen bzw. behandeln können.

### Regenerative Peripheral Nerve Interface

Das Konzept des Regenerative Peripheral Nerve Interface (RPNI) beschreibt den Transfer eines kleinen denervierten und avaskulären Muskelstücks auf ein distales Nervenende im Amputationsstumpf (Abb. 3d). Die Technik wurde initial in Kombination mit implantierten Elektroden im Tiermodell vorgestellt, um Muskelsignale nachzuweisen, welche für die Prothesensteuerung verwendet werden können [23]. Es konnte gezeigt werden, dass das transferierte Muskelgewebe reinnerviert wird und langfristig als Signalgeber dienen kann. Inzwischen wurden auch schon zwei Patienten mit transradialer Amputation präsentiert, welche mittels RPNI und intramuskulären Elektroden eine Handprothese mit selektiver Aktivität einzelner Finger steuern konnten [36]. Um mehr Signale zu ermöglichen bzw. den Axonen mehr Muskelgewebe zur Reinnervation zu bieten, werden die einzelnen Nerven interfaszikulär gespalten, um mehrere getrennte Nervenenden zu erzeugen [19]. Für jede Faszikelgruppe wird dann ein eigenes kleines Muskeltransplantat mittels nichtresorbierbarer Nähte fixiert und schließlich um den Nerv gewickelt fixiert. Die notwendigen Muskeltransplantate können entweder von der amputierten Extremität oder einem großen Spendermuskel wie dem Vastus lateralis des M. quadriceps femoris entnommen werden. Die Muskelstücke werden so platziert, dass die Muskelfasern parallel zu den Axonen zu liegen kommen.

Wie auch bei TMR hat sich bei RPNI gezeigt, dass neben den funktionellen Aspekten auch positive Effekte auf Postamputationsschmerzen erzielt werden können. In einer retrospektiven Analyse verglichen Kubiak et al. 45 Patienten mit RPNI während der Amputation mit 45 Patienten, welche unterschiedliche nervenchirurgische Verfahren erhielten, unter anderem alleinige Rückkürzung, Ligatur der Nerven oder intramuskuläre Transposition [20]. Sie konnten für die RPNI-Gruppe eine deutlich niedrigere Inzidenz von schmerzhaften Neuromen (0 % vs. 13 %) sowie ebenso eine niedrigere Prozentzahl an Phantomschmerzen (51 % vs. 91 %) feststellen. Auch für Neurome im Bereich der Hand konnten positive Ergebnisse bei 85 % der Patienten erzielt werden [16]. Zuletzt stellte dieselbe Arbeitsgruppe im Tiermodell ein kombiniertes RPNI vor, welches neben Muskel auch Hautgewebe enthält, um somit sensible Rückmeldung in das Konzept zu integrieren [33].

## Diskussion

Es ist allgemein akzeptiert, dass Nerven im Rahmen einer Amputation rückgekürzt werden sollen, um weit entfernt von der Oberfläche und von Gelenken platziert zu werden. Dies geschieht mit dem Ziel, sie vor Narbenbildung sowie mechanischem Stress weitgehend zu schützen. Darüber hinaus besteht Konsens, dass schmerzhafte Stumpfneurome reseziert werden müssen, um diese zu therapieren. Die Frage stellt sich schließlich, welches Verfahren das ideale im Umgang mit dem Nervenende nach Neuromresektion ist.

Grundsätzlich haben sich durch Rückkürzung und intramuskuläre Verlagerung historisch gute Ergebnisse in Bezug auf Neuromschmerzen zeigen lassen. Jedoch gilt es bei intakter Extremität, insbesondere bei motorischem Schaden oder Verlust kritischer Sensibilität, weiterhin als Goldstandard, den Nerv wenn möglich zu rekonstruieren. Guse und Moran haben 2013 in einer retrospektiven Analyse die Ergebnisse von Nervenrekonstruktion, Nerventransposition und alleiniger Rückkürzung des Nervs bei Neuromen der oberen Extremität verglichen [14]. Es zeigten sich bessere Ergebnisse in Bezug auf allgemeine Einschränkung der Armfunktion und Reoperationsrate für die Rekonstruktionsgruppe, während von alleiniger Rückkürzung aufgrund der hohen Rezidivrate abgeraten wurde. Die Möglichkeit der direkten Rekonstruktion bietet sich bei Amputationen nicht an, da die distalen Nervenenden bzw. deren Endorgane nicht mehr vorhanden sind.

Mit der Entwicklung von TMR konnte erstmals auch Stumpfnerven denerviertes Muskelgewebe zur Reinnervation angeboten werden. Die hiermit neu geschaffenen intuitiven Myosignale können zu einer verbesserten Steuerung von myoelektrischen Prothesen verwendet werden. Besonders bei hohen Amputationen ist TMR zu diesem Zweck inzwischen etabliert. In Bezug auf Schmerzen scheint TMR einer alleinigen Rückkürzung und Verlagerung des distalen Nervensegments überlegen zu sein, was in der bisher einzigen randomisiert-kontrollierten Studie zu dieser Thematik nachgewiesen wurde. Insbesondere die Ergebnisse zu Phantomschmerzen sind sehr überzeugend, da zu dieser komplexen Problematik bisher noch kaum positive Effekte durch chirurgische Eingriffe bekannt waren. Die Überlegenheit von TMR gegenüber konventionellen Methoden wird häufig dadurch erklärt, dass man den regenerierenden Nervenfasern sinnvolle, funktionelle Endorgane bietet und diese somit zur Ruhe kommen. Eine Limitation dieser Technik ergibt sich durch die unweigerlich längere Operationszeit, weshalb viele Chirurgen, die Autoren eingeschlossen, bei primären Amputationen noch davon absehen – so keine funktionelle Indikation gegeben ist.

Bezüglich der verwandten TSR-Methode gibt es noch wenige publizierte Erfahrungsberichte. Es ist zu erwähnen, dass im Bereich der Stumpfhaut nur eine limitierte Anzahl an Hautnerven zur Verfügung steht, was die Anwendung dieser Technik einschränkt. Außerdem kommt es nach Durchtrennen eines sensiblen Hautastes, was im Rahmen dieser Methode notwendig ist, unweigerlich zu einem neuen Neurom, welches langfristig Schmerzen verursachen kann. Weiters ist die entstehende Sensibilität nach Reinnervation eines Hautareals mit kognitiv ektopen Nervenfasern nicht dem natürlichen Tastsinn der verlorenen Extremität gleichwertig. Es kann dabei, ähnlich wie auch nach Rekonstruktion sensibler Nerven, zu unangenehmen Missempfindungen (Parästhesien) kommen, welche oft als Kribbeln oder Ameisenlaufen beschrieben werden.

Wie auch TMR bietet RPNI dem Nerv denerviertes Muskelgewebe, in welchem die regenerierenden motorischen und gegebenenfalls auch sensiblen Nervenfasern neue Endorgane finden. Die praktische Umsetzung des funktionellen Potenzials dieser Technik für die myoelektrische Prothetik konnte zwar inzwischen demonstriert werden, benötigt jedoch implantierbare Elektroden und findet daher bisher nur im Rahmen klinischer Forschungsprojekte Anwendung. Die Ergebnisse zu Phantom- und Neuromschmerzen sind, ähnlich wie bei TMR, vielversprechend, mit dem Vorteil, dass die operative Technik einfacher zu erlernen ist. Jedoch stellt sich zumindest theoretisch die Frage, ob durch kleine Muskelstücke einem großen Nerv genügend funktionelle Endorgane zur Reinnervation geliefert werden, oder ob der Effekt zur Neuromprophylaxe nicht schlussendlich ein ähnlicher wie bei der intramuskulären Transposition ist. Auch ist es nicht klar, für welchen Zeitraum solche avaskulären Muskelgewebe im Menschen vital bleiben bzw. ob es nicht langfristig zu einer Vernarbung kommen kann.

Somit kann abschließend festgehalten werden, dass die Amputationschirurgie heute weit mehr Möglichkeiten als noch zu Anfang dieses Jahrhunderts bietet. Die Autoren bevorzugen bei „Standard“-Amputationen der unteren Extremität die Rückkürzung der Nerven mit Verlagerung in einen tiefen Muskel, möglichst weit entfernt von einer Belastungszone oder einem Gelenk. Vor Rückkürzung erfolgt das Einspritzen des proximalen Nervensegments mittels Lokalanästhetikum, um eine Fortleitung des Schmerzreizes nach zentral zu verhindern. Wenn bei hohen Amputationen der oberen Extremität eine Versorgung mittels myoelektrischer Prothese und mehr als zwei Signalen geplant ist, kommt TMR zum Einsatz. Für die übrigen Fälle gelten für den Umgang mit Nerven die gleichen Prinzipien wie an der unteren Extremität. Bei Neuromschmerzen ist eine Resektion des Neuroms auf jeden Fall notwendig. Zur reinen Schmerztherapie ist dann in den meisten Fällen zwar eine intramuskuläre Verlagerung erfolgreich, jedoch sollten hier TMR bzw. auch RPNI als sinnvolle Alternativen erwogen werden, insbesondere in Anbetracht der rezenten positiven Studienergebnisse. Auch bzgl. der Phantomschmerzen bieten beide Techniken durch den Effekt der Reafferenzierung einen theoretischen Vorteil, welcher sich in den entsprechenden Studien ebenfalls zeigen ließ. Um mehr Klarheit in der Indikationsstellung dieser Techniken zu schaffen, wären für die Zukunft weitere prospektive, randomisiert-kontrollierte Langzeitstudien wünschenswert, um die Effekte von intramuskulärer Transposition, TMR und RPNI auf Neurom- und Phantomschmerzen zu vergleichen. Hinsichtlich der funktionellen Aspekte nervenchirurgischer Operationen am Stumpf wird sich durch den potenziellen zukünftigen Einsatz von implantierbaren elektronischen Systemen in der klinischen Routine auch eine entsprechende Anpassung der chirurgischen Vorgehensweise ergeben.

## Fazit für die Praxis

- Neuromprophylaxe ist bei der initialen Amputation wichtig, in der Regel mittels hoher Neurotomie und Verlagerung des Nervenendes in tiefe Muskulatur, entfernt von Gelenken.
- Neben der Klinik sind hochauflösender Ultraschall, MRT und selektive Nervenblockaden zur Diagnose von Stumpfneuromen hilfreich.
- Schmerzhafte Neurome sollten reseziert werden, die Autoren anästhesieren das proximale Nervensegment vor der Neurotomie.
- Neuromresektion und Implantation von Nerven in einen tiefen Muskel führt im Großteil der Fälle zu einem guten Ergebnis.
- Für Targeted Muscle Reinnervation (TMR) gibt es inzwischen sehr gute Evidenz zu positiven Effekten auf Neurom- und Phantomschmerzen, mit Überlegenheit zu einfacher Implantation in Muskel, jedoch längerer Operationsdauer.
- TMR sollte durchgeführt werden, wenn mehr Myosignale zur Prothesensteuerung notwendig sind, vor allem bei glenohumeralen und transhumeralen Amputationen.
- Das Regenerative Peripheral Nerve Interface ist eine relativ neue Technik, welche auch gute Ergebnisse in Bezug auf Neurom- und Phantomschmerzen liefert.

## Abkürzungen

NRS: Numeric Rating Scale
RPNI: Regenerative Peripheral Nerve Interface
TMR: Targeted Muscle Reinnervation
TSR: Targeted Sensory Reinnervation
